# How Does the Location of Transfer Affect Travellers and Their Choice of Travel Mode?—A Smart Spatial Analysis Approach

**DOI:** 10.3390/s20164418

**Published:** 2020-08-07

**Authors:** Jason Chia, Jinwoo (Brian) Lee, Hoon Han

**Affiliations:** 1 School of Civil Engineering and Built Environment, Science and Engineering Faculty, Queensland University of Technology, Brisbane, QLD 4000, Australia; jasonchiaqut@gmail.com; 2 City Planning, City Planning Discipline, School of Architecture and Built Environment, Faculty of Built Environment, The University of New South Wales, Sydney, NSW 2052, Australia; h.han@unsw.edu.au

**Keywords:** transit, bus, transfer, smart card, spatial analysis, mode choice

## Abstract

This study explores the relationship between the spatial distribution of relative transfer location (i.e., the location of the transfer point in relation to the trip origin and destination points) and the attractiveness of the transit service using smart card data. Transfer is an essential component of the transit trip that allows people to reach more destinations, but it is also the main factor that deters the smartness of the public transit. The literature quantifies the inconvenience of transfer in terms of extra travel time or cost incurred during transfer. Unlike this conventional approach, the new “transfer location” variable is formulated by mapping the spatial distribution of relative transfer locations on a homogeneous geocoordinate system. The clustering of transfer points is then quantified using grid-based hierarchical clustering. The transfer location factor is formulated as a new explanatory variable for mode choice modelling. This new variable is found to be statistically significant, and no correlation is observed with other explanatory variables, including transit travel time. These results imply that smart transit users may perceive the travel direction (to transfer) as important, in addition to the travel time factor, which would influence their mode choice. Travellers may disfavour even adjacent transfer locations depending on their relative location. The findings of this study will contribute to improving the understanding of transit user behaviour and impact of the smartness of transfer, assist smart transport planning and designing of new transit routes and services to enhance the transfer performance.

## 1. Introduction

Smart, reliable and connected service has been a long-standing goal of transit agencies. Smart transit network and service design must consider the service connectivity to allow users to travel to spatially diverse destinations [1,2]. However, providing direct connectivity for all origin—destination pairs is simply infeasible and impractical. Smart transfer is becoming an essential component of the transit trip. The extra effort in making transfers efficient and convenient has deemed to be necessary to expand service coverage and to provide competitive area-wide connectivity [1,2]. Ironically, transfer is recognised by travellers as a significant impeding factor that disrupts the transit travel experience and deters the use of transit [3,4,5]. The literature formulates the effect of transfer in terms of the extra travel time such as the additional walking time, waiting time and in-vehicle travel time, incurred during transfer [5,6,7]. Another type of transfer penalty encapsulates subjective and psychological factors based on preferences, attitudes and perceptions of transit users [1,3,8].

The literature formulates the effect of transfer in the scalar form such as the extra walking time, waiting time, in-vehicle travel time and monetary transfer cost incurred during transfer [5,6,7]. Another type of transfer penalty encapsulates subjective and psychological factors based on preferences, attitudes and perceptions of transit users [1,3,8].

This study builds on the hypothesis that transfer location (i.e., the relative location of the transfer point to the trip origin and destination) has an impact on the attractiveness of the transit trip involving the transfer for travellers. This variable is quantified by the level of deviation of the transfer point from the straight path from the trip origin to destination. The deviation could be interpreted as intrinsic factors that reflect subjective or psychological impedance imposed by the transfer location. Whereas the burden of transfer has been quantified in terms of travel time and/or cost in the literature, we propose a new “transfer location” variable to represent the deviation in the travel direction. We examine if transit users tend to disfavour the transfer locations that deviate from a direct path to the trip destination and test this hypothesis by incorporating this novel explanatory variable to represent its underlying effect on the travel mode choice. To control the distance effect, this study proposes a combination of transformation techniques to keep only the deviation of transfer points. Despite an extensive range of research on transfer, the current literature has neglected the potential implication of transfer location in the decision-making of mode choice.

In the present study, we aim to improve the smartness of transfer and the explanatory ability of mode choice models by incorporating the transfer location variable. The findings of this study could contribute to smart transport planning and designing of new transit routes and smart services to enhance the transfer quality and to a more realistic assessment of transit service accessibility and connectivity. The spatial distribution of transfer points is analysed by their relative location with respect to the destination point. This study presents a transformation approach to convert the actual coordinates of the transit journey itineraries (i.e., origin, destination and transfer points) on a two-dimensional homogeneous geocoordinate. This approach may be useful for transit route choice and further transfer location analysis.

## 2. Literature Review

Transfer is an essential and inevitable component of the transit journey (a chain of trips). It allows passengers to reach more destinations by switching to different routes and modes, hence enhances the smartness of the transfer. In major cities with a multimodal transit system, the role of the transfer is more prevalent. In an integrated transit system, the focus is to provide seamless transfers between different trips in a journey [2,9]. Smart transfers at strategic locations improve transit connectivity and expand spatial coverage of transit systems [10]. Despite its essential role, transfers are often seen as a burden in using transit [11]. Inconvenient transfers deter the use of transit for potential transit users and reduce the satisfaction level of existing transit users, which ultimately leads to reduction in the ridership.

The conventional way of quantifying the inconvenience of transfer has been by incorporating it into a generalised cost term to account for the extra monetary costs, travel time and discomfort incurred during the service transferring [5,12,13]. Transfer penalty can be measured as an equivalence of the travel time or money saving by taking the ratio between the coefficients of transfer variables and time or cost variables. This ratio shows how much further people are willing to travel (time without transfer) or how much they are willing to pay (cost), to save one transfer, demonstrating the time and money that must be saved in order to justify one transfer [3,5]. The literature suggests that out-of-vehicle travel time is perceived as more onerous than in-vehicle travel time by transit users when making transfers [14,15]. In practice, the general rule of thumb is that walking and wait times are valued twice as much as the in-vehicle travel time [7,12]. Wardman, et al. (2001) suggest that bus users value the wait time about 1.2 times higher than the in-vehicle travel time and the walk time 1.6 times higher than the in-vehicle travel time [7]. Generally, the wait time during transfer is also valued higher than the walking time during transfer [12,16].

Operational factors such as service reliability, headways regularity, on-time performance and the availability of adequate information affect the quality of transfers [5,12,17]. Providing a guaranteed connection and a through ticket for transfer could significantly reduce the perceived penalty of transfers [7]. An empirical study conducted in Haifa, Israel demonstrated that waiving a transfer fee resulted in a significant increase in the transit ridership [6]. Another study conducted in metropolitan Los Angeles showed that the users’ satisfaction with the transit service transfer has little to do with the physical characteristics of the facility, but service frequency and reliability have more impact [18]. A study by Currie and Loader found that the volume of transfers could significantly increase along a major transit route when the service headway is 10 min or shorter [2].

Physical environmental factors such as stop and station amenities may affect the smartness of transfer services. Guo and Wilson reported that transit users are more likely to use the transfer service if escalators are available at the transfer station to assist with changing of levels [11]. Providing amenities such as benches, shades, water fountains and rest rooms would increase the comfort and convenience of transit users while waiting and transferring [5,12]. Security and safety, such as the presence of security staffs and the actual crime rates within the transit facilities would affect the perception of transfer quality [19]. A case study of the London underground train found that the worst transfer locations were the stations with the largest and most complex transfer environments, and the best transfer locations perceived were those stations with simple transfer environments [20]. In the case of whether to take a transfer or walk a longer distance to a destination, Guo and Wilson found that the demand of transfer decreases if walking environments are improved [11]. For example, if wider sidewalks exist along the non-transfer path, transit users are less likely to use a transfer service.

In an integrated transit system, more research seeks to understand and minimise the real cost of transfer inconvenience [15]. Much effort has been devoted to understand and minimise the cost of different time components (e.g., walk and wait time) during transfer, such as the timed transfer concept. This concept optimises the slack time between the arrival of incoming vehicles with the departure of outgoing vehicles [21,22,23]. Ceder et al. developed a synchronised timetable by maximising the number of simultaneous bus arrivals at transfer nodes [14]. Shih et al. employed the heuristic model for the design of a coordinated network with transfer centres [24]. Similarly, Ting and Schonfeld used a heuristic algorithm to optimise the headways and slack times jointly for all coordinated routes, as the optimised slack times vary with different variables such as headways, vehicle arrival time variance and transfer volumes [25].

As much as minimising the transfer time is important, transit users could also consider the travel direction towards the transfer point. Conventionally, the inconvenience to transfer caused by transfer location is considered as an increase in transit travel time, in the scalar form. This concept is similar to the “angular cost” concept presented by Raveau et al. to measure the directness of a chosen transit route [26]. The conventional route choice models account for the service level of the route alternatives and the socioeconomic and demographic characteristics of users [27]. Raveau et al. found that transit users tend to penalise routes that deviate from a direct path to their destination [26]. The “angular cost” is measured as a function of $\;\sin\left(\frac{\theta }{2}\right)$, where θ is the angle formed between the direct path to the destination (*OD*) with the origin-transfer (*OT*) straight route, weighted by the Euclidean distance to transfer point (d).

## 3. Study Area and Data

The city of Brisbane accounts for approximately 70% of the total daily weekday trips in South East Queensland [28]. Brisbane has an extensive transit network of bus, rail and ferry systems, covering more than 10,000 km^2^. The recent report by the Queensland Government revealed that from January to March 2016, 27.38 million trips were conducted by bus, followed by 12.21 million trips by train, 1.71 million trips by ferry and 1.93 million trips by tram [29]. Bus ridership consisted of more than 63% of total transit ridership. This shows that the bus is the dominant transit mode in Brisbane. The benefit of the bus, in comparison to the train, tram and ferry, is that it has the flexibility to access almost all locations where a road network is present. The nature of buses travelling on existing road networks gives more feasibility of adapting to change, such as the addition of new bus routes to serve more destinations. These considerations have steered the scope of this research towards bus ridership in Brisbane.

Brisbane’s bus network may be characterised as a typical radial structure where more than 66% of the bus services operating to the city centre [30]. There are many routes heading in the same direction with very minor variations and no feeder or trunk services are currently provided. The CBD is the central hub for the bus system, where three grade-separated bus only corridors (busways) provide high-speed, high-capacity services to regional centres.

This study relies on two main data sources. First is the smart card data (big data), which is used to develop the transfer map of bus users in the study area. The one-day “go-card” data of Brisbane (24 November 2014, Monday) was used for the mapping. The data encapsulates the entire Brisbane area. The go-card is an electronic ticket for use on transit services throughout the network and records travel data when a traveller touches on at the start of any trip stage, and touches off at the end of the trip stage. This dataset contains information such as go-card ID, date of service, route ID, service ID, direction (inbound or outbound), boarding time and alighting time, boarding stop ID and alighting stop ID, ticket type, journey ID and trip ID. If it is a transfer journey, it would have a consecutive trip ID for each trip stage with the identical journey ID. According to TransLink, a journey is defined as the set of trip stages taken under one fare basis, while a trip is a ride on a single transit vehicle. This study adopts the same convention for the terms “journey” and “trip”.

The second dataset used in this research is the 2009 Southeast Queensland Household Travel Survey (SQHTS). This single cross-sectional survey provides information on daily travel behaviour of all members of participating households, from 20 April through 28 June 2009. This includes how and why they travel, at what time of day journeys are made and the average journey distance and duration [28]. Respondents were also asked to report a range of personal information (e.g., age, gender, individual income, driver’s license, etc.), and household related information (e.g., household size, number of vehicles, etc.).

## 4. Transformation Mapping of Transfer Coordinate

This section presents a transformation approach to project the transfer locations on a homogeneous coordinate to examine the spatial distribution pattern of transfers. Travellers could be guided by the geographical images formed in minds, rather than the external maps, especially when individuals are familiar with the settings. These mental constructions suffice as the sole source of spatial information. At instances when individuals are unfamiliar with the surroundings and need to rely on an external map, they still have to transcribe the cartographic information into their minds before they can act on the information [31,32]. In both instances, it is the spatial images in minds that best explain travellers’ spatial behaviours.

### 4.1. Processing for Single-Transfer Journey Itineraries

**Figure 1:**
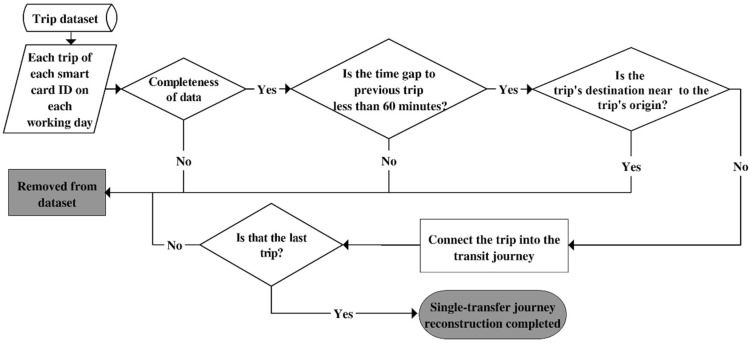
Process to construct single-transfer travel journeys.

The smart card data was processed to filter out direct bus journeys and the journeys with two or more service transfers. The single transfer bus journeys account for about 20% of the total bus journeys. The journeys with two or more transfers are negligible less than 1% of the total bus journeys. The analytical framework of the transfer impact in this paper was developed applicable to only single-transfer journeys. The first step of the transformation mapping is to reconstruct travel itineraries by combining related trips from each smart card holder to form complete journeys from origins to destinations, including transfers. The data processing to construct single-transfer journeys is shown in Figure 1.

The process starts by filtering out noise data such as the incomplete data of origin or destination information. A threshold of 60-min time gap (from the time when travellers alight a stop, to their next boarding time) is applied to identify whether two transactions are connected as a transfer journey. A different threshold has been chosen differently in the literature, ranging from 30 to 90 min [33,34], or a set of thresholds for different transit modes [35]. The threshold of the 60-min time gap is recommended in accordance with Brisbane’s transit authority, based on the observed transfer behaviour and transit service characteristics [29]. If the transit user stays at a place for more than 60 min before making the next trip, those two trips are counted as separate trips, rather than a continuous journey through a transfer.

The next process is to distinguish return trips from single-transfer journeys. Studies have shown that transit users are willing to walk on average 400 or 500 m to bus stops [36,37,38,39]. A maximum distance threshold of 1 km from origin and destination was used to distinguish single-transfer journeys from return trips. To illustrate, the first bus stop could be located 500 m to the left of the journey’s origin (e.g., the residence) and the last bus stop could be located 500 m to the right of the journey’s destination (e.g., the residence). If the first and last bus stops are located less than 1 km apart, for the purpose of this study, it was assumed to be a return trip. This study was only interested in single-transfer journeys, so if there was any journey that had more than one transfer, the whole journey was removed from the dataset. After the reconstruction process, a total of 10,083 journeys were identified.

### 4.2. Transformation

**Figure 2:**
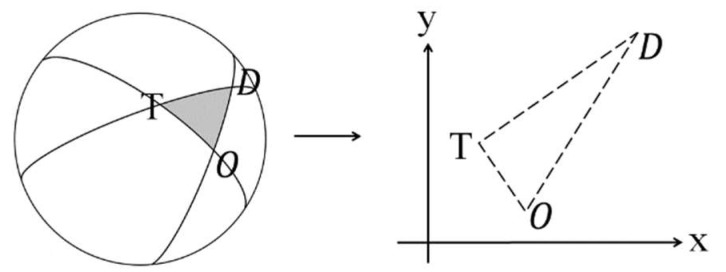
Transformation from a spherical Earth’s surface to a 2D plan.

The transit journey data may be illustrated as a triangle where each point of triangle represents the coordinate of the trip origin, destination and transfer. The size of the journey triangles varies by the actual trip distance and therefore the journey data needs to be converted into a homogeneous coordinate system to analyse the spatial distribution pattern. The conversion is done by applying a series of transformation techniques in this study. The first step of the transformation is to transform the journey triangle *OTD* (origin–transfer–destination) on a spherical Earth’s surface to a 2D plan, given the latitudes and longitudes of each point of interest, as shown in Figure 2.

The great-circle distance between two points, which is the shortest distance over the Earth’s surface, is calculated based on the spherical law of cosines. The spherical law of cosines states that, for a spherical triangle,

(1) cosOD=cosOTcosTD+sinOTsinTDcosT

where,

O,T,D = Interest points of the journey triangle, *OTD*
OD = Distance between origin point and destination point
OT = Distance between origin point and transfer point
TD = Distance between transfer point and destination point

The location of any point on the earth can be defined by its latitude and longitude. In reference to Equation (1), the *OD* distance can be calculated as the arccosine of cos *OD*, as shown in Equation (2).

(2) $$\begin{array}{l} \cos OD=\cos OT\cos TD+\sin OT\sin TD\cos T\\ OD\;\left(\mathrm{in}\;\mathrm{rad}.\right)=\cos^{-1}\;[\cos OT\cos TD+\sin OT\sin TD\cos T] \end{array}$$

The unit used for angles is in radians, which gives the distance between origin and destination in radians. Given the convenient mean radius of the earth to be equivalent to 6371 km, the distance between origin and destination, in km, can be calculated by multiplying the *OD* distance (in radians) with 6371 km, as shown in Equation (3).

(3) $$OD\;\left(\mathrm{in}\;\mathrm{km}\right)=\cos^{-1}\;[\cos OT\cos TD+\sin OT\sin TD\cos T]*6371\;\mathrm{km}$$

The same technique was applied to calculate the great-circle distance of *OT* and *TD*. With the great-circle distance of *OT*, *TD* and *OD*, the respective angles of any triangle on a 2D plane could be calculated using the law of cosines, as shown in Equation (4).

(4) $$\begin{array}{l} \cos O=(OT^{2}+OD^{2}-TD^{2})/2\left(OT*OD\right)\\ O={\;\cos}^{-1}\left[(OT^{2}+OD^{2}-TD^{2}\right)/2\left(OT*OD\right)] \end{array}$$

**Figure 3:**
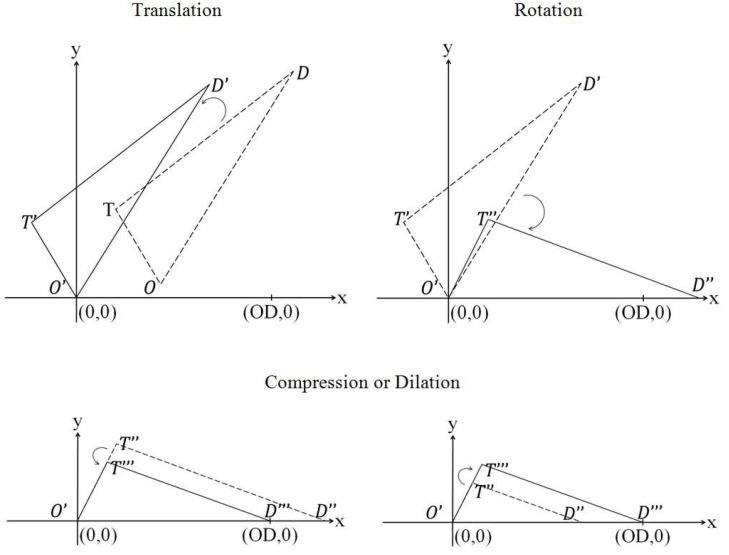
Euclidean transformations.

After the journey triangle *OTD* was obtained, it needs to undergo a series of Euclidean transformations to display all the origin, destination and transfer points in a standardised Euclidean space, as illustrated in Figure 3.

The first step of Euclidean transformation is translation. Translation relocates the journey triangle *OTD* to set the triangle’s origin point, *O*, at (0, 0). This transformation preserves the congruence and distance of the journey triangle *OTD*. Applying the translation process to the single-transfer journeys results in all the journey triangles originating from the same point at (0, 0). The notation for translation ($T_{h,k}$) is shown in Equation (5). The origin and destination points will undergo the same transformation.

(5) $$T_{h,k}\;\left({T^{\prime }}_{x},{T^{\prime }}_{y}\right)=\left(T_{x}+h,\;T_{y}+k\right)$$

Preserving the congruence and distance, the journey triangle *OTD* is rotated at *O* (0, 0) until the triangle plane, *OD*, rests on the *x*-axis. This transformation rotates all the journey triangles to lie along the *x*-axis for the destination point, *D*, to have the coordinate of (*x*, 0). The notation for rotation is shown in Equation (6).

(6) $$\left[\begin{array}{c} {T^{\prime \prime }}_{x}\\ {T^{\prime \prime }}_{y} \end{array}\right]=\left[\begin{array}{cc} \cos\theta & -\sin\theta \\ \sin\theta & \cos\theta \end{array}\right]\left[\begin{array}{c} {T^{\prime }}_{x}\\ {T^{\prime }}_{y} \end{array}\right]$$

**Figure 4:**
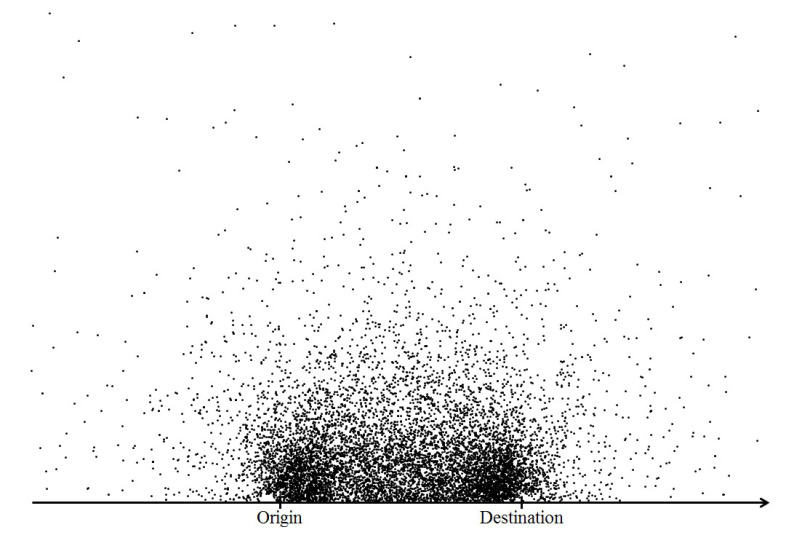
Transfer locations on a homogeneous coordinate.

At this stage, all journey triangles *OTD* lie on the same plane (*x*-axis). The next step of the transformation is to loosen up the restriction to consider bijection, which preserves the shape and angles of the triangle, but not distance. The aim of this step is to transform all journey triangles *OTD* to have the same *OD* unit distance, as shown in Figure 4. The notation for compression and dilation ($CD_{k}$) is shown in Equation (7).

(7) $$CD_{k}\;\left({T^{\prime \prime \prime }}_{x},{T^{\prime \prime \prime }}_{y}\right)=\left(k{T^{\prime \prime }}_{x},k{T^{\prime \prime }}_{y}\right)$$

### 4.3. Transfer Location Map

Figure 4 illustrates the transfer points of the single-transfer bus journeys, transformed to the scale of *OD* unit distance for both the *x* and *y* axis. In the figure, the scale is not the actual distance, but is adjusted through either compression or dilation. This study assumes that the plot represents the “acceptable” or “viable” transfer locations in relation to the straight path to destination. Consequent analysis quantifies and ranks the viability of transfer points and validates its impact on the travel mode choice.

The distribution of transfer points in Figure 4 may have been influenced by the availability and quality of transit services in the area. They are chosen transfer locations possibly among multiple alternatives, available to the travellers. The transit network structure must be an important determinant of the distribution pattern. The transit network of Brisbane takes the typical radial structure, with no trunk or feeder service. It is common that a transfer requires a significant deviation from the direct path to the destination or even in the opposite direction from the destination. The distribution pattern in the figure may reflect the inconvenience factor of the service transfer under the existing network structure.

### 4.4. Grid-Based Hierarchical Clustering

**Figure 5:**
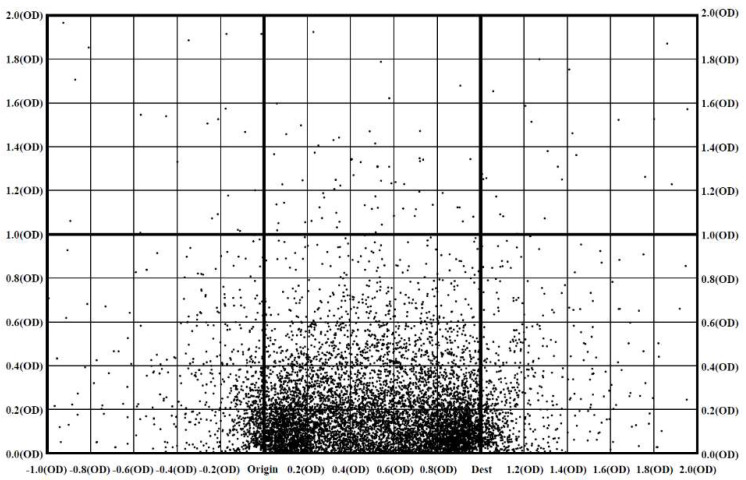
Grid structure on the transformed transfer location map.

To analyse the spatial distribution pattern of the transfer points, this study used the grid-based hierarchical clustering method, which combines the grid-based clustering and hierarchical clustering methods. Cluster analysis is a data reduction tool that partitions a sample dataset into clusters, where objects within a specific cluster share many characteristics, but are very dissimilar to objects not belonging to that cluster [40]. The grid-based clustering (also known as density-based clustering) is one of the most efficient approaches for mining large data sets. Unsupervised clustering such as K-means was inappropriate for this study because it clusters the data in similar sizes (i.e., point densities). The underlying assumption of the choice modelling in the next step is that the cells with higher point densities are considered as more viable transfer locations by travellers. This method adopts algorithms that partition the data space into a finite number of cells to form a grid structure [41] as shown in Figure 5.

For grid-clustering, each grid is defined as 0.2 × *OD* (origin to destination) unit distance increment. These transfer points are plotted in reference to 1.0 × *OD* unit distance. Figure 5 shows the clear concentration of transfer points in the cells, along with the direct path between the origin and destination. For cell clustering, the cell density is calculated for each cell as follows:

(8) $$\mathrm{Cell}\;\mathrm{density}=\frac{\mathrm{Total}\;\mathrm{number}\;\mathrm{of}\;\mathrm{transfer}\;\mathrm{points}\;\mathrm{in}\;\mathrm{grid}\;x}{\mathrm{Total}\;\mathrm{number}\;\mathrm{of}\;\mathrm{transfer}\;\mathrm{points}}$$

**Figure 6:**
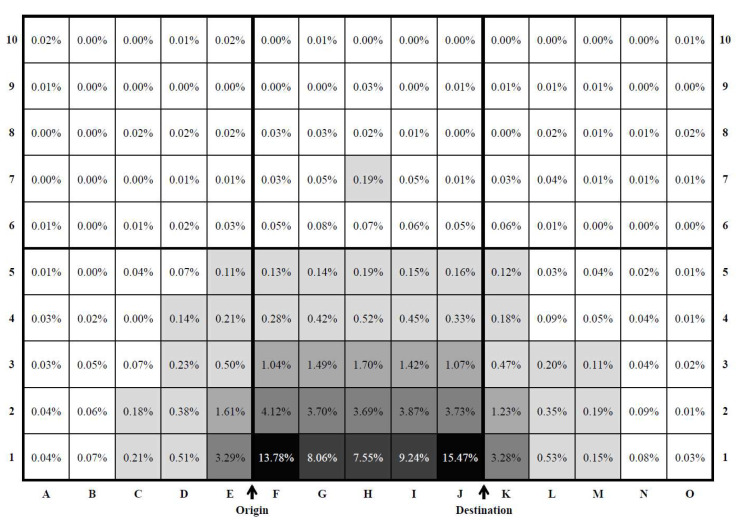
Cell-density in respective clusters.

The hierarchical clustering method was applied to sort the cells into clusters. Hierarchical clustering is useful for finding relatively homogenous clusters of cases based on measured characteristics. It starts with each case as a separate cluster. Next, these clusters are combined sequentially until only one cluster is left. The algorithm for this clustering method uses the dissimilarities or distances between objects when forming the clusters [40]. Figure 6 shows the cell-density for each cell, and to which cluster each cell is assigned by the hierarchical clustering method.

**Figure 7:**
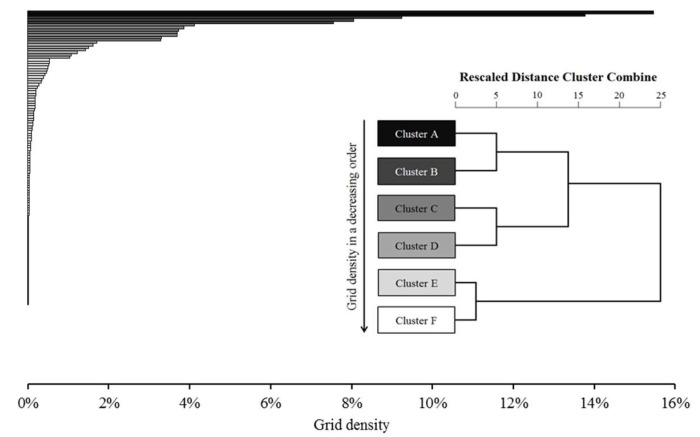
Cell density dendrogram.

Figure 6 presents the preferred transfer locations with relatively high cell densities. Figure 7 shows the result of hierarchical clustering. Some interesting results were observed in the travellers’ transfer selection. The majority of bus journeys conducted had made a transfer located in the cells F1 and J1. These two cells were identified to have the highest transfer point density at 13.78% and 15.47%, respectively (Cluster A). These two cells may be regarded as the most preferred transfer location and by having a transfer service in those cell locations will increase the likelihood of making a transfer and eventually taking a transit, compared to other cells.

All the other cells are categorised into five different clusters by the cell’s grid density. The hierarchical clustering uses the Ward’s method to measure the dissimilarity among clusters. Ward’s method uses an analysis of variance approach, instead of distance metrics to evaluate the distances between clusters, where cluster membership is assessed by calculating the total sum of squared deviations from the mean of a cluster [42]. The dendrogram allows the tracing backward and forward to any cluster at any level. It gives an idea of how great the distance is between clusters in a particular step, using the 0–25 scale along the top of the chart.

Cluster B includes G1, H1 and I1. Transfer points in those five cells of Cluster A and Cluster B account for 54.1% out of the total 150 cells in the map. This implies that most travellers prefer the transfer point to be closely located along the direction to their trip destination. Cluster C consists of seven cells, E1, F2 to J2 and K1. The average cell density significantly declined to 3.67%. Some bus users travelled to transfer points in the opposite direction from their destination, but not too far from their origination location. Similarly, some travellers made a transfer farther from their destination location. The cell density further decreased for the cells in Cluster D with the average density value at 1.37%. The transfer points located in the Cluster A to D groups accounted for 89.35% of the total transfers. The average density value of the cells in Cluster E and Cluster F was negligible at 0.27% and 0.02%, respectively although they accounted for more than 87.33% of the total map area (131 out of 150 cells).

In general, based on the transfer point density in each cell, it was observed that the majority of transfers are conducted at the locations along the direct path to the destination. Transit users occasionally travel to the opposite direction from the destination, or slightly farther away from the destination to make a transfer. When a transit journey is required to make a transfer that is deviated from the direct path between origin and destination, the realisation of such trips is unlikely. This demonstrates the impedance of transfer location, and the interpretation must take into account the transit network structure.

## 5. Mode Choice Analysis

Two binomial logistic regression models (base and expanded model) are drawn on two travel modes: private vehicles and the bus. For a mode choice analysis, the information of mode specific variables of the alternative (unchosen) mode is necessary. Information of the alternative mode only can be inferred. This study used the GTFS (General Transit Feed Specification) data to infer the bus journey information for those who have chosen a private vehicle as their travel mode; and Google Maps to infer the private vehicle travel time for those who have chosen the bus as their travel mode. This analysis considers only the home-based work journeys. If a traveller has used the bus as the mode of transport, the journey must include one service transfer using the same travel mode (bus). Due to the strict criteria, only 330 private vehicle journeys and 63 bus journeys were used for the analysis. The 2009-10 SEQHTS is the most recent and detailed dataset available to demonstrate travellers’ travel patterns and mode choice.

The dependent variables of the model are dichotomous, representing travel mode choice (transit or private vehicle). The independent variables tested in this analysis include individual characteristics (gender, age, individual weekly income, household size and number of cars in the household), journey attributes (travel time, initial wait time, first mile walk time and last mile walk time) and transfer attributes (proportion of in-vehicle bus travel time, proportion of transfer walk time, proportion of transfer wait time, type of transfer and transfer location). Table 1 presents the list of independent variables with brief descriptions.

Two different models were developed to test the effectiveness of the transfer location variable. The base model (Model I) takes the conventional approach to account for the effect of transfer by incorporating the proportion of the in-vehicle bus travel time, proportion of transfer walk time and proportion of transfer wait time variables. The expanded model (Model II) used the same set of independent variables and an additional the “transfer location” variable. The test results of those two models are presented in Table 2.

Table 2 shows only the variables that provided the best fitting model fit. For instance, gender, age, network distance, transfer walking time, transfer wait time and transfer type were found not to be significant. The best-fitting basic model (Model I) incorporated six independent variables including: individual weekly income, household size, number of cars, car travel time, bus travel time, initial wait time for the first bus service, first mile walk time, last mile walk time and proportion of in-vehicle bus travel time. In Model II, the transfer location variable was found significant at the 0.05 level. This is notable as the new variable was found to make substantial influence on the mode choice, significant at the 0.05 level. As for socioeconomic variables, the household size had a positive effect on the utility of transit, whereas the individual weekly income and number of cars in the household had a negative effect on the transit utility. The car travel time factor was found significant (at the 0.01 level) among other journey attributes. Other journey attributes such as the first mile and last mile walk time were found significant at the 0.1 level for both the base and expanded models, which are consistent with the literature. As the access and egress increases, the use of transit decreases [38,43]. The initial wait time for the first bus service was found significant at the 0.1 level, only for the expanded models. If transit is not available at the time when individuals needed to travel, it decreases the attractiveness of transit.

As for the transfer-related variables, only the proportion of the in-vehicle bus travel time factor was found to be significant in both the base and expanded models (significant at the 0.05 level). Exp. *β* shows the effect of the independent variable on the odds ratio. The Exp. *β* coefficient relating the proportion of in-vehicle transit travel to the likelihood of using transit was 49.45 and 69.27 in the base and expanded model, respectively. These results implied that travellers are more likely to use transit as the proportion of in-vehicle bus travel time increases. This finding is consistent with the literature that shorter in-vehicle transit travel times could lead travellers to perceive the walking and wait times during transfer more onerous and eventually increases the relative attractiveness of a private vehicle [1,20,44,45].

The transfer location variable in the expanded model was found to be significant at the 95% confidence level. The negative coefficient suggests that a transfer location farther from the OD path will decrease the utility of bus and the probability to choose the bus mode. In fact, it turns out that the transfer location factor is one of the most important determinants of travel mode choice. This variable has the Exp. *β* (the odds ratio) value of 0.75, which shows that a change in the transfer location from a more preferred cluster to a less preferred cluster (e.g., from Cluster A to Cluster B) would decrease the probability of choosing the bus to 0.43, and increase the probability of choosing a private vehicle to 0.57.

The prediction capability of Model I and Model II was compared using McFadden rho squared to demonstrate the effectiveness of the new transfer location variable and its impact on the travel mode choice. Model I resulted in the pseudo R-squared, *ρ*^2^ at 0.29, whereas Model II increased it to 0.31. McFadden suggested *ρ*^2^ values of between 0.2 and 0.4 should represent a very good fit of the model [46]. The increase in *ρ*^2^ by Model II demonstrates that with the inclusion of the new variable, Model II has a better explanatory power on mode choice as compared to Model I.

The chi-squared (χ^2^) test was conducted to investigate the statistical improvement between Model I and Model II, by gauging the change in the log-likelihood function relative to the change in degrees of freedom. The chi-squared, χ^2^ value of 5.04 exceeds the critical chi-squared of 1 degree of freedom of 3.84, at the 0.05 significant level. This gives a sufficient evidence to reject the null hypothesis that Model II is no better than Model I. With the inclusion of the transfer location variable into Model II, it outperforms Model I (base model).

The transfer location variable in Table 2 is ordered from the most preferred cluster to the least preferred cluster, in an ordinal-scale. This approach is effective to study the impact of transfer location as a variable, based on the assumption that the distance between clusters is equal. To study the relationship between the clusters, an additional binomial logistic regression model is conducted to include the transfer location variable as nominal variables. The result is shown in Table 3.

The result from Table 2 and Table 3 did not differ much. The age of the travellers became significant at the 0.05 confidence level, with a negative effect on transit utility. Having the transfer location as nominal variables, Cluster F was assigned to be the reference category. The exponential *β* coefficient shows that if a transfer location is in Cluster A, it will have 5.99 times more chance to use the bus over Cluster F. Transfer locations located in Cluster B, C and D were found to be significant at the 0.05 level, but not Cluster E. This implies that as the transfer location changes from a less preferred cluster to a more preferred cluster (e.g., from Cluster F to Cluster A), it will increase the probability of choosing the bus over an automobile.

### Transfer Location and Transit Travel Time

The analysis results indicate that the chance to make a transit trip was likely to decrease if the trip involves a transfer at the location that deviates from the direct path to the destination. In the conventional mode choice analysis, the level of deviation is quantified in terms of travel time incurred during the transfer. The new variable was created to capture the impact of deviation in the travel direction. We take two approaches to examine the potential collinearity between transfer location and transit travel time. Firstly, the Spearman’s correlation coefficient (*ρ*) was calculated between the bus travel time (continuous variable) and the transfer location (categorical variable) of the Household Travel Survey bus trip data. A weak correlation (*ρ* = −0.211) was found between two variables, which implies that the location of transfer may play as an independent factor for the travel mode choice.

**Figure 8:**
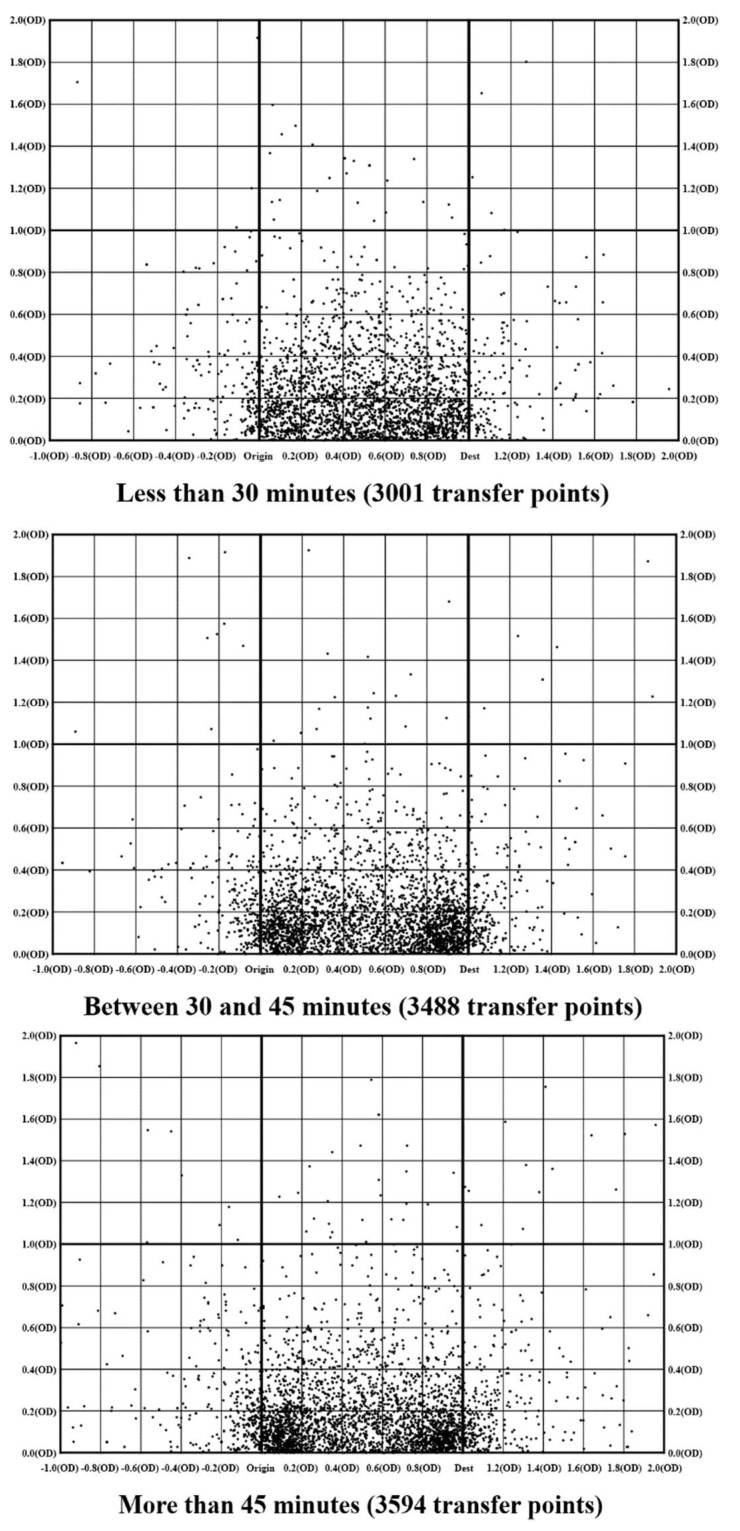
Distribution of transfer points.

The second approach presents three plots of the transfer point distribution by the length of the bus journey time (less than 30 min, between 30 and 45 min and more than 45 min), as shown in Figure 8.

The level of deviation of transfer points was derived using Equation (9). An arbitrary cell length of 4 was used—for example, the deviation of a transfer point (*x*, *y*) was calculated as the sum of the distance from the origination point (0, 0) and the distance from the destination point (20, 0).

(9) $$\mathrm{Level}\;\mathrm{of}\;\mathrm{deviation}=\sqrt{x^{2}+y^{2}}+\sqrt{{\left(x-20\right)}^{2}+y^{2}}$$

The average level of deviation of a short (less than 30 min), medium (between 30 and 45 min) and long journey (longer than 45 min) was found at 24.7, 24.3 and 25.7, respectively. Although more deviation was found among the longer bus trips, the distribution pattern is largely unchanged regardless of the length of travel time. This suggests that the preference for transfer location was not affected by the travel time (or distance) and travellers might disfavour adjacent transfer services depending on their relative location with respect to destination. The conventional approach of using door-to-door travel time to capture the transfer cost is not sufficient. Transit travel time may be able to capture the effect of deviation in the travel distance, but it is not capable to capture the effect of deviation in the travel direction towards transfer services.

## 6. Conclusions

This paper proposed a new approach to take into account the smart distribution of the transfer location impact on travel mode choice. A transformation method was proposed for mapping of the transfer locations on a two-dimensional homogeneous geocoordinate. Transformed transfer locations were grouped into six classes by the level of point density. The novel transfer location variable was included in a mode choice model to demonstrate its underlying effect. The new variable was found as one of the driving determinants of mode choice. The study revealed that the transfer services in the “preferred locations” are likely to increase the smartness of the transit journey including the transfer. The transfer location and the in-vehicle travel time variables were found to be significant and uncorrelated to each other. This implies that travel direction towards transfer points may be an important factor pertaining to mode choice in addition to the travel time factor. The conventional approach of using door-to-door travel time may be not sufficient to capture the real cost of transfer.

This study provides a new approach to analyse the spatial distribution of transfer locations in relation to trip origin and destination points. This new geocoordinate technique may be useful for many applications in smart transport research including transit accessibility and connectivity studies. The conventional methods define the transit service accessibility and connectivity using a travel time constraint, where accessible areas by transit are simply defined as the travel boundary within a specific travel time period (e.g., 45 min). In a radial transit network structure, travelling to neighbouring suburbs often require a transfer at the opposite direction from the destination if there is no direct transit route connecting two suburbs, which is not so smart. For choice users (a private vehicle is available), such locations may be deemed as inaccessible by using transit. Integrating the transfer location variable to the traditional accessibility and connectivity measures may provide a more realistic representation of the service coverage of transit systems.

The findings of this study may contribute to improving the smartness of the public transit and the prediction capability of the mode choice analysis for the future transport demand. The findings presented in this study should be viewed as an exploratory effort to developing a new approach to account for the smartness of transfer and to test is effect on mode choice. The main findings will assist the transit service and performance assessment to identify service gaps and underserved areas. Identifying convenient and strategic transfer locations is essential so that scarce resources can be channelled effectively to improve the quality and smartness of transit service. Minimising the perceived transfer penalty will assist in increasing the competitiveness of public transport, and eventually the transit ridership. In this study, the emphasis is only given to bus journeys with a single transfer. Future research could build upon this concept to consider multimodal transit journeys and those journeys with more than a single transfer.

## Figures and Tables

**Table 1:** List of independent variables.

Variable	Description
**Socioeconomic Attributes**	
Gender	Nominal variables: 0—male; 1—female
Age	Age of individuals
Individual weekly income	Individuals’ weekly income, given in different income bracket
Number of cars	Total number of cars per household
Household size	Number of persons in the household
**Journey Attributes**	
Car travel time (minutes)	Total time taken to travel from origins to destinations using private vehicle
Bus travel time (minutes)	Total time taken to travel from origins to destinations using bus
Initial wait time (minutes)	Total wait time for the next available bus service
First mile walk time (minutes)	Walk time taken to access bus station from origination
Last mile walk time (minutes)	Walk time taken from bus station to destination
**Transfer Attributes**	
Proportion of in-vehicle bus travel time	Proportion of a journey spent on two buses
Proportion of transfer walk time	Proportion of a journey spent on walking for a transfer
Proportion of transfer wait time	Proportion of a journey spent on waiting for a transfer
Type of transfer	Nominal variables: 0—non-walking transfer; 1—otherwise
Transfer location	Ordinal and nominal variables: The cluster developed using smart card data (i.e., Cluster A–F encoded to 0–5), of which individual transfer location falls into

**Table 2:** Binomial logit model results: transfer location as an ordinal variable.

Variables	Model I			Model II		
	Base Model			Expanded Model		
	Coefficient	Std. Err.	Exp. *β*	Coefficient	Std. Err.	Exp. *β*
Constant	−0.85	1.27	0.43	0.35	1.39	1.42
**Socioeconomic Attributes**						
Individual weekly income	−0.00 ***	0.00	1.00	−0.00 ***	0.00	1.00
Household size	0.43 ***	0.14	1.54	0.40 ***	0.14	1.49
Number of cars	−1.22 ***	0.24	0.29	−1.31 ***	0.25	0.27
**Journey Attributes**						
Car travel time (minutes)	0.04 ***	0.02	1.04	0.06 ***	0.02	1.07
Initial wait time (minutes)	−0.03	0.02	0.97	−0.03 *	0.02	0.97
First mile walk time (minutes)	−0.08 *	0.04	0.93	−0.09 **	0.04	0.92
Last mile walk time (minutes)	−0.07 *	0.04	0.93	−0.08 **	0.04	0.92
**Transfer Attributes**						
Proportion of in-vehicle bus travel time	3.90 **	1.54	49.45	4.24 **	1.59	69.27
Transfer location	Not included			−0.28 **	0.13	0.75
Number of observation			393			393
Log-likelihood function value: Constant only model			−172.99			−172.99
Log-likelihood function value: Parameterised model			−122.40			−119.88
Goodness of fit (McFadden rho squared)			0.29			0.31
Model Improvement Test: −2 *(log-likelihood of basic model—log-likelihood of expanded model)						5.04
Chi-critical based on 1 degree of freedom						3.84

Notes: ***: *p* < 0.01; **: *p* < 0.05; *: *p* < 0.1. Coefficients that are statistically insignificant (*p* ≥ 0.1) are not shown in this table.

**Table 3:** Binomial logit model results: transfer location as nominal variables.

Variables	Model I			Model II		
	Base Model			Expanded Model		
	Coefficient	Std. Err.	Exp. *β*	Coefficient	Std. Err.	Exp. *β*
Constant	0.16	1.37	1.18	−0.98	1.53	0.38
**Socioeconomic Attributes**						
Age	−0.03 **	0.01	0.97	−0.03 ***	0.01	0.97
Individual weekly income	−0.00 ***	0.00	1.00	−0.00 ***	0.00	1.00
Household size	0.34 **	0.14	1.41	0.32 **	0.15	1.37
Number of cars	−1.32 ***	0.24	0.27	−1.48 ***	0.26	0.23
**Journey Attributes**						
Car travel time (minutes)	−0.05 ***	0.02	0.95	−0.07 ***	0.02	0.94
Initial wait time (minutes)	−0.02 *	0.01	0.98	−0.02 **	0.01	0.98
Last mile walk time (minutes)	−0.07 *	0.04	0.93	−0.08 **	0.04	0.92
**Transfer Attributes**						
Proportion of in-vehicle bus travel time	4.00 ***	1.51	54.52	4.52 ***	1.59	91.84
**Transfer Location** **The reference category: Cluster F**						
Cluster A	Not included			1.79 *	1.05	5.99
Cluster B				2.34 **	0.98	10.40
Cluster C				2.31 **	0.96	10.04
Cluster D				1.99 **	1.01	7.30
Cluster E				1.21	0.95	3.36
Number of observation			393			393
Log-likelihood function value: Constant only model			−172.99			−172.99
Log-likelihood function value: Parameterised model			−121.58			−116.20
Goodness of fit (Nagelkerke R Square)			0.39			0.43
Goodness of fit (McFadden R Square)			0.30			0.33
Model improvement test (Chi-squared test, χ^2^): −2 *(log-likelihood of basic model—log-likelihood of expanded model)						10.76
The critical chi-squared value with 5 degrees of freedom at the 0.10 α-level						9.24
The critical chi-squared value with 5 degrees of freedom at the 0.05 α-level						11.07

Notes: ***: *p* < 0.01; **: *p* < 0.05; *: *p* < 0.10. Coefficients that are statistically insignificant (*p* ≥ 0.10) are not shown in this table.
